# The Danger of Living in Glass Houses

**Published:** 1896-06

**Authors:** 


### THE DANGER OF LIVING IN GLASS HOUSES.

No country has placed more embargoes on our animal food-products than Germany; yet of 50 preparations sold in their markets for coloring and preserving meats 27 contained boric acid. Of 154 samples of butter examined, no less than 45 were adulterated, and yet they have repeatedly charged this country with sending them impure food-products, and placed obstacles in the way of the sale of our animal food-products that would lead one to believe that the supervision and examination on their own soil were so complete and perfect that only pure foods could be sold on their markets. People who live in glass houses should not throw stones, and there will henceforth be a stronger belief in the fact that our food-products are kept out because they are better and cheaper than their own.

